# Perioperative management of critical obstetric hemorrhage after cesarean delivery in a patient with hereditary hemorrhagic telangiectasia: a case report

**DOI:** 10.1186/s40981-025-00821-9

**Published:** 2025-10-21

**Authors:** Saori Hayashi, Ryo Wakabayashi, Ken Kobayashi, Junko Tsukamoto, Kazuhito Mietani, Kanji Uchida

**Affiliations:** Department of Anesthesiology and Pain Relief Center, The University of Tokyo Hospital, 7-3-1, Hongo, Bunkyo-ku, Tokyo 113-8655 Japan

**Keywords:** Cesarean delivery, Critical obstetric hemorrhage, Hereditary hemorrhagic telangiectasia, Multidisciplinary management, Postpartum hemorrhage

## Abstract

**Background:**

Hereditary hemorrhagic telangiectasia (HHT) is a rare autosomal dominant vascular disorder associated with hemorrhagic complications. In pregnant women with HHT, postpartum hemorrhage can be life-threatening and may necessitate hysterectomy.

**Case presentation:**

A 36-year-old primigravida woman with HHT underwent emergency cesarean delivery due to nonreassuring fetal status. Following delivery, she developed massive uterine bleeding. Despite intrauterine balloon tamponade, hemorrhage persisted. Under the direction of the attending anesthesiologist and obstetrician, maternal resuscitation was initiated. Contrast-enhanced computed tomography revealed active extravasation from the right uterine artery. During emergent interventional radiology, angiography identified contrast extravasation from a hypertrophied spiral artery at the peripheral end of the right uterine artery, and embolization achieved initial hemostasis. She subsequently received intensive care under the supervision of the critical care team and ultimately recovered without hysterectomy.

**Conclusions:**

Rapid multidisciplinary intervention for critical obstetric hemorrhage in a patient with HHT led to maternal survival and uterine preservation.

## Background

Hereditary hemorrhagic telangiectasia (HHT), also known as Rendu–Osler–Weber syndrome or Osler’s disease, is an autosomal dominant vascular disorder characterized by abnormal angiogenesis and arteriovenous malformations, with a prevalence of 1 in 2,000 to 40,000 individuals [1]. It is caused by mutations in genes encoding components of the bone morphogenetic protein (BMP) signaling pathway, such as *ENG*, *ACVRL1*, *SMAD4*, and *GDF2* [2], resulting in defective formation of the elastic and muscular layers of blood vessels and increased vascular fragility [3].

Pregnancy-related hemorrhagic complications in HHT are typically associated with arteriovenous malformations of the lungs, liver, or central nervous system [4]. By contrast, in patients with HHT, severe postpartum hemorrhage (PPH) following cesarean delivery—potentially requiring hysterectomy—has been only sporadically reported and remains poorly documented [5].

The present report describes a case of massive PPH following cesarean delivery in a patient with HHT, in which rapid multidisciplinary intervention enabled uterine preservation despite life-threatening bleeding. Written informed consent was obtained from the patient for publication.

## Case presentation

The patient was a 36-year-old primigravida woman (gravida 1, para 0) with a height of 161 cm and a weight of 70 kg (pre-pregnancy weight: 48 kg). She had been diagnosed with HHT during pregnancy based on the Curaçao criteria, fulfilling three major components: recurrent epistaxis, telangiectasia of the nasal mucosa, and a family history of HHT in her mother. Genetic testing for HHT was not performed. Prenatal imaging revealed no vascular malformations in the lungs, liver, or central nervous system. Standard laboratory tests showed a hemoglobin level of 12.3 g/dL, platelet count of 172,000 /mm^3^, prothrombin time–international normalized ratio of 1.24, activated partial thromboplastin time of 32.9 s, and fibrinogen level of 322 mg/dL (Table 1**)**.

**Table 1:** Results of standard laboratory testing throughout the clinical course

Timing	Hemoglobin (g/dL) (11.6–14.8)	Platelet count (/mm^3^) (158,000–348,000)	PT-INR (0.85–1.15)	APTT (s) (24.0–34.0)	Fibrinogen (mg/dL) (167.7–354.7)
Baseline	12.3	172,000	1.24	32.9	322
After cesarean delivery	9.4	107,000	1.69	60.0	149
After CT	6.8	71,000	-^a^	-^a^	-^a^
During IVR	7.8	38,000	1.84	59.2	107
ICU admission	8.1	74,000	1.46	43.6	247
3 h after ICU admission	7.1	57,000	1.44	37.7	227
6 h after ICU admission	7.9	81,000	1.29	35.4	286
9 h after ICU admission	9.2	67,000	1.27	34.6	289
12 h after ICU admission	12.7	56,000	1.25	32.7	345
24 h after ICU admission	12.2	76,000	1.39	32.5	367
2 days after ICU admission	12.1	78,000	1.47	34.0	421
3 days after ICU admission	12.3	95,000	1.45	32.5	532
4 days after ICU admission	12.3	102,000	1.28	31.2	502

Values in parentheses indicate reference ranges established by our institution

*APTT* activated partial-thromboplastin time, *CT* computed tomography, *ICU* intensive care unit, *IVR* interventional radiology, *PT-INR* prothrombin–time international normalized raito

^a^Measurement could not be performed due to unsuitable sample volume

At 38 weeks and 3 days of gestation, an epidural catheter was placed for labor analgesia. Artificial rupture of membranes was performed at 38 weeks and 5 days. Labor was induced at 38 weeks and 6 days; however, due to meconium-stained amniotic fluid and prolonged decelerations observed on cardiotocography, which led to a diagnosis of intrauterine infection with nonreassuring fetal status, the patient underwent emergency cesarean delivery.

Upon entering the operating room, the epidural catheter was removed, and spinal anesthesia was administered using 2.4 mL of 0.5% hyperbaric bupivacaine combined with 15 μg of fentanyl. Two 20-gauge peripheral intravenous catheters were used for intraoperative fluid management. The sensory block level was confirmed by cold test to be at the fourth thoracic dermatome on the left and the fifth thoracic dermatome on the right.

The neonate was delivered 5 min after the start of surgery, and the placenta was delivered 1 min later. Apgar scores were 7 and 9 at 1 and 5 min, respectively, and the umbilical arterial pH was 7.235. To promote uterine contraction, 10 units of oxytocin were administered intravenously mixed in an infusion bottle, and 5 units intramyometrially as directed by the attending obstetrician. When the uterus became atonic again during uterine closure, the same doses were re-administered via the respective routes. The initial shock index was 0.8 and increased to 1.1 by the end of the procedure. The surgery was completed in 73 min, with blood loss of 750 mL.

Postoperative vaginal examination performed in the operating room revealed active hemorrhage from the uterine cavity. Despite intravenous bolus administration of 0.2 mg methylergometrine, bleeding persisted. In coordination with the attending anesthesiologist and obstetrician, an 18-gauge peripheral venous catheter and a 22-gauge radial arterial catheter were placed. Simultaneously, rapid colloid infusion was initiated, 1 g of tranexamic acid was administered intravenously, and blood sampling was performed along with the ordering of pre-arranged blood products.

**Fig. 1:**
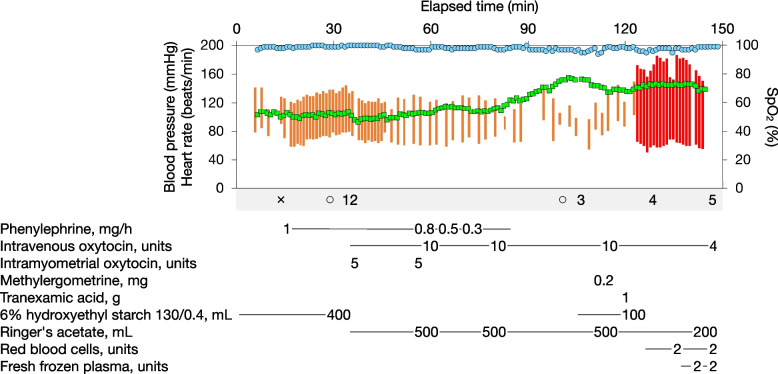
The course of vital signs and major drug, fluid, and transfusion records obtained in the operating room. The orange line indicates noninvasive blood pressure (systolic to diastolic range), the red line invasive blood pressure (systolic to diastolic range), the green squares heart rate, and the blue circles oxygen saturation measured by pulse oximetry (SpO₂). Remark symbols are as follows: × , spinal anesthesia; ○, start and end of surgery; 1, delivery of the neonate; 2, placental delivery; 3, postoperative vaginal examination; 4, intrauterine balloon tamponade; and 5, transfer to the computed tomography room

Despite intrauterine balloon tamponade, hemorrhage persisted, and total blood loss reached 2,550 mL. Given a shock index exceeding 1.5 and laboratory findings indicative of coagulopathy—specifically, a hemoglobin level of 9.4 g/dL, platelet count of 107,000 /mm^3^, prothrombin time–international normalized ratio of 1.69, activated partial thromboplastin time of 60.0 s, and fibrinogen level of 149 mg/dL—a diagnosis of critical obstetric hemorrhage was made, and a critical obstetric hemorrhage protocol [6] was activated. The radiology department was promptly contacted to prepare for emergency contrast-enhanced computed tomography (CT), with interventional radiology (IVR) to be performed contingent upon the findings. Figure 1 illustrates the course of vital signs recorded in the operating room.

**Fig. 2:**
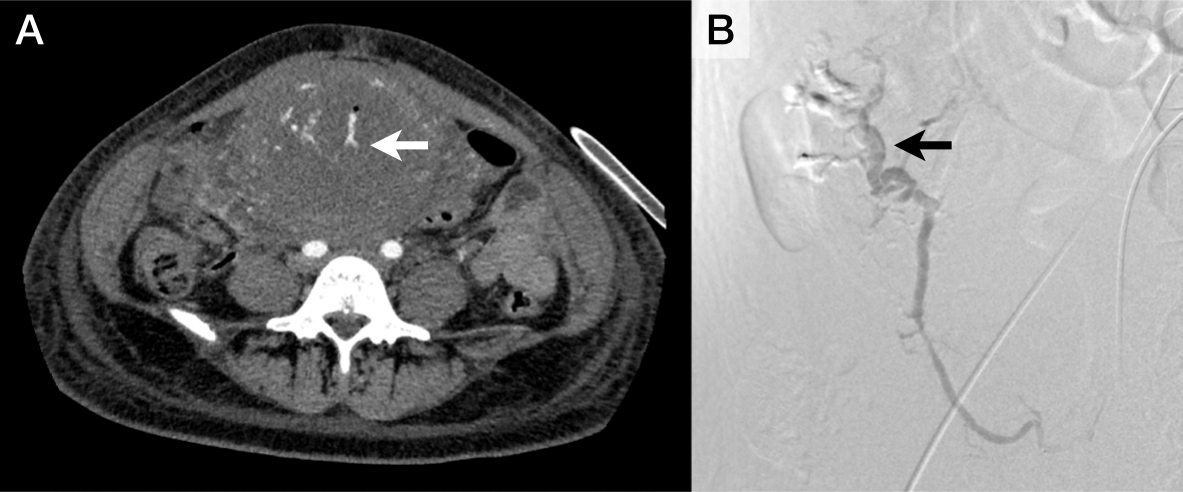
Imaging findings of arterial contrast extravasation. **A** Axial contrast-enhanced computed tomography at the level of the fifth lumbar vertebra, showing contrast extravasation from the right uterine artery (arrow). **B** Angiographic image obtained during interventional radiology, indicating contrast extravasation from a hypertrophied spiral artery at the peripheral end of the right uterine artery (arrow)

While receiving transfusion of four units of packed red blood cells (RBC) and four units of fresh frozen plasma (FFP), the patient was transferred for CT under continuous hemodynamic monitoring. CT revealed active extravasation from the right uterine artery (Fig. 2A), and the critical care team was subsequently engaged to support maternal resuscitation. Post-CT laboratory data demonstrated a hemoglobin level of 6.8 g/dL and a platelet count of 71,000 /mm^3^, prompting transfusion of an additional four units of RBC and four units of FFP.

Emergency IVR revealed contrast extravasation from a hypertrophied spiral artery at the peripheral end of the right uterine artery (Fig. 2B**)**, and timely embolization was performed using a gelatin sponge. During IVR, 16 units of RBC, 24 units of FFP, 20 units of platelet concentrate, 32 units of cryoprecipitate, and 3 g of fibrinogen concentrate were administered.

Although initial hemostasis was achieved with IVR, minor but persistent bleeding continued, resulting in an estimated total blood loss exceeding 6,000 mL. The obstetric disseminated intravascular coagulation score was calculated as 9 [7]. Accordingly, critical care was continued in the intensive care unit (ICU) under the supervision of intensivists. In the ICU, the patient received 28 units of RBC, 42 units of FFP, 30 units of platelet concentrate, and 3 g of fibrinogen concentrate. Hemodynamic stability was achieved, and anemia and coagulopathy gradually improved over the clinical course (Table 1). The patient was also treated for pulmonary congestion and sepsis, and was discharged from the ICU on postoperative day 4, followed by discharge from the hospital on day 21. Throughout the perioperative course, tracheal intubation and mechanical ventilation were not required. Post hoc analysis of maternal blood samples obtained after cesarean delivery revealed activation of the complement system (Table 2).

**Table 2:** Results of post hoc analysis of the complement system

C3 (mg/dL) (80.0–140.0)	C4 (mg/dL) (11.0–34.0)	C1 esterase inhibitor activity (%) (≥ 42.0)
56.0	4.0	26.0

Values in parentheses indicate reference ranges provided by the external laboratory

## Discussion

PPH remains a leading cause of pregnancy-related mortality worldwide, accounting for approximately one-quarter of maternal deaths [8]. Its etiology is commonly categorized by the mnemonic “Four Ts”: tone (uterine atony), trauma (lacerations, hematomas, inversion, or rupture), tissue (retained products of conception or abnormal placentation), and thrombin (coagulopathies) [9]. To reduce maternal morbidity and mortality, prompt identification of the underlying cause and timely intervention are imperative [10]. In the present case, the massive hemorrhage was deemed primarily attributable to uterine atony and subsequent coagulopathy.

Complement activation detected in maternal blood samples and postoperative pulmonary congestion suggest that amniotic fluid embolism (AFE) may also have contributed [11]. However, a definitive diagnosis could not be established, partly because hysterectomy was avoided and therefore histopathological confirmation was not possible. The combination of uterine atony, persistent coagulopathy, and pulmonary congestion nevertheless supports consideration of this mechanism [12].

In addition, the bleeding was resistant to uterotonics and balloon tamponade and demonstrated arterial contrast extravasation, findings that share some similarities with reports of PPH with resistance to conventional treatments and arterial contrast extravasation (PRACE) [13, 14]. As PRACE is not yet widely accepted, these similarities should be regarded as supplementary rather than definitive evidence.

From a pathophysiological standpoint, impaired BMP-mediated vascular remodeling associated with HHT could plausibly have contributed to hypertrophy and fragility of the spiral arteries in this patient [15, 16]. However, because hysterectomy was avoided, histopathological confirmation could not be obtained, and further studies are needed to validate this mechanism.

Regarding anesthetic and obstetric management, the epidural catheter was removed and spinal anesthesia was selected to provide rapid and reliable anesthesia under emergent conditions. The intrathecal dose may have been higher than typically recommended for parturients with prior labor epidural analgesia [17], but no high spinal block occurred. Oxytocin was administered as 10 units intravenously (mixed in an infusion bottle) and 5 units intramyometrially, with repeat dosing when uterine atony recurred, at the request of the attending obstetrician. This relatively high-dose regimen [18] may have necessitated additional large-volume infusion through the vasodilatory effects of oxytocin, particularly in the context of ongoing hemorrhage, thereby contributing to dilutional coagulopathy. Earlier administration of methylergometrine might have been more appropriate once the initial response proved inadequate. Preoperatively, two large-bore venous lines were secured and blood products were prepared in anticipation of the increased risk of massive hemorrhage in a patient with HHT. Intraoperatively and postoperatively, anesthesiologists, obstetricians, radiologists, and intensivists collaborated closely to provide transfusion support, IVR, and intensive care. These aspects highlight both the limitations and strengths of management in this case and emphasize the importance of preparedness and multidisciplinary coordination [19].

In conclusion, we present a case of massive PPH following cesarean delivery in a patient with HHT, managed with rapid and coordinated multidisciplinary care, resulting in maternal survival and uterine preservation. This case underscores the importance of careful preoperative planning, intraoperative vigilance, and timely collaboration across specialties when managing hemorrhagic complications in patients with HHT undergoing cesarean delivery.

## Data Availability

The datasets used and/or analyzed during the current study are available from the corresponding author on reasonable request.

## Abbreviations

AFE: Amniotic fluid embolism
BMP: Bone morphogenetic protein
CT: Computed tomography
HHT: Hereditary hemorrhagic telangiectasia
IVR: Interventional radiology
PPH: Postpartum hemorrhage
PRACE: Postpartum hemorrhage with resistance to conventional treatments and arterial contrast extravasation
